# Bioavailability of Supplemented Free Oleanolic Acid and Cyclodextrin–Oleanolic Acid in Growing Pigs, and Effects on Growth Performance, Nutrient Digestibility and Plasma Metabolites

**DOI:** 10.3390/ani14192826

**Published:** 2024-09-30

**Authors:** Manuel Lachica, Isabel Borrás-Linares, Thays Helena Borges, Rosa Nieto, Isabel Seiquer, Consolación García-Contreras, Luis Lara, David Arráez-Román, Antonio Segura-Carretero, José María Pinilla, José Carlos Quintela, Ignacio Fernández-Fígares

**Affiliations:** 1 Department of Nutrition and Sustainable Animal Production, Estación Experimental del Zaidín, CSIC, San Miguel 101, Armilla, 18100 Granada, Spain; manuel.lachica@eez.csic.es (M.L.); rosa.nieto@eez.csic.es (R.N.); iseiquer@eez.csic.es (I.S.); consolacion.garcia@eez.csic.es (C.G.-C.); llara@eez.csic.es (L.L.); 2 Research and Development Functional Food Centre (CIDAF), Edificio Bioregión, Avenida del Conocimiento, 37, Armilla, 18016 Granada, Spain; iborras@ugr.es; 3 Department of Analytical Chemistry, Faculty of Sciences, University of Granada, Fuentenueva s/n, 18071 Granada, Spain; darraez@ugr.es (D.A.-R.); ansegura@ugr.es (A.S.-C.); 4 Natac Biotech S.L.U. Rita Levi Montalcini 14, Getafe, 28906 Madrid, Spain; jmpinilla@natacgroup.com (J.M.P.); jcquintela@natacgroup.com (J.C.Q.)

**Keywords:** bioavailability, digestibility, growth, oleanolic acid, pig

## Abstract

**Simple Summary:**

Oleanolic acid is an organic natural compound, abundant in olive leaves, with various beneficial health effects in humans and animals. However, its in vivo efficacy is questioned given its low solubility, which hinders its bioavailability, that is, the capacity of a molecule to reach circulation. We investigated the digestibility and plasma concentration of oleanolic acid as an estimate of bioavailability in growing pigs. Because it is important to know the effects of oleanolic acid in the animal, growth, organ weights, digestibility of nutrients and plasma biochemical profile have been reported as well. Although there is a concern in the scientific community regarding the low bioavailability of oleanolic acid, in vivo data are lacking. We have demonstrated that, while digestibility of oleanolic acid was unexpectedly elevated, the appearance of the molecule in systemic blood was weak, probably indicating hepatic metabolism. No negative effects of oleanolic acid on growth or internal organs were observed.

**Abstract:**

Oleanolic acid (OLA) has beneficial health effects in animals, but in vivo efficacy in monogastric animals is questioned due to its low bioavailability. To gain further insight on the nutritional effects of OLA it was administered as part of a diet. We investigated digestibility and plasma OLA in pigs and the associated influence on growth, organs, digestibility of nutrients and plasma biochemical profile. Twenty-four crossbred barrows (23.7 ± 1.0 kg BW) were assigned one of three treatments: Control (basal diet without OLA), OLA-1 (basal diet with 260 mg/free OLA) and OLA-2 (basal diet with 260 mg/kg cyclodextrin-OLA). Diets included chromium oxide to estimate digestibility. Blood samples were collected on day 14 for OLA analysis and feces on days 22–24 for determining digestibility. Pigs were slaughtered on day 31 (39.9 ± 2.43 kg BW) and their blood collected for analysis. Growth and organ weights were not affected (*p* > 0.05). OLA-1 decreased apparent total tract digestibility (ATTD) of energy (*p* < 0.05). OLA-2 increased ATTD of dry and organic matter compared with Control pigs (*p* < 0.05). OLA-1 increased plasma calcium and alkaline phosphatase (*p* < 0.05). Ileal digestibility of OLA was not affected (0.88), although OLA ATTD increased in OLA-1 compared to Control pigs (0.75 vs. 0.82; *p* < 0.05). OLA-1 and OLA-2 increased plasma OLA compared to Control pigs (*p* < 0.05 and *p* = 0.083). In conclusion, although the OLA was digested and absorbed, plasma concentration was low (4.29 µg/L), and pig growth, organs and plasma parameters were not affected.

## 1. Introduction

Oleanolic acid is a pentacyclic triterpenoid abundant in plants of the Oleaceae family, mainly Olea europaea, from which its name is derived. Olive tree by-products, such as leaves, contain significant amounts of oleanolic acid (above 3% dry weight [1,2,3]), and consequently, they are used commercially as source for oleanolic acid extraction. It has been reported that oleanolic acid has beneficial biological effects, including improvement of gut atrophy and microbiota [4,5], immunomodulatory effects [6], anti-inflammatory/antioxidant effects [7], anticarcinogenic effects [8], antiatherogenic effects [9], retardation of atherosclerosis [10], antidiabetic effects [7,11] hepatoprotective effects [12] and neuroprotective effects [13]. Furthermore, epidemiological studies have established that the traditional Mediterranean diet is, worldwide, amongst the healthiest diets, and this has been linked to the consumption of abundant amounts of olive oil [14]. Hence, oleanolic acid is receiving attention on account of the potential benefits of its consumption as a therapeutic agent or dietary supplement. Biological effects of food components do not depend solely on their concentration, but rather on their bioavailability, which is the proportion that enters the systemic circulation when consumed and is available at the target tissue. The bioavailability of oleanolic acid, both with Caco-2 cells and in vivo, was recently reviewed [15]. Oleanolic acid has shown a low oral bioavailability in rodents, which may be partly explained by its low aqueous solubility and poor intestinal permeability [16,17,18]. One important factor affecting bioavailability is related to the physicochemical properties of the molecule, as oleanolic acid has high lipophilicity [15] and low wettability [17]. Thus, oleanolic acid absorption has been hampered by poor dissolution and slow partitioning between the cell membrane and extracellular fluids in rats [17]. Accordingly, different approaches have been described in the literature as aiming to increase the limited solubility of oleanolic acid, such as the use of nanosuspensions [13], spray freeze dryers, micro- and nanoemulsions [19,20], or different formulations of compounds conjugated with oleanolic acid [20,21,22]. Some of these preparations could have an impact on the digestibility of oleanolic acid. In the present research, we have made use of oleanolic acid’s digestibility and its appearance in systemic blood after oral intake as a proxy to estimate the bioavailability of oleanolic acid. Furthermore, in vivo studies to ascertain bioavailability of oleanolic acid are lacking, particularly in large animals, so another innovation of this investigation is the use of growing pigs. We hypothesized that the presumed weak bioavailability of oleanolic acid was not due to the low digestibility of the compound itself. Therefore, the aims of the present study were, first, to evaluate the digestibility of oleanolic acid at both the fecal and the ileal level, and to determine the systemic blood appearance after oral ingestion in growing pigs. To this end, we have measured oleanolic acid digestibility and its appearance in systemic blood after oral intake as a proxy to estimate the bioavailability of oleanolic acid. The second objective was to determine whether oleanolic acid intake affected growth parameters, digestibility of nutrients, and the plasmatic biochemical profile of growing pigs. Additionally, the outcomes of this study could be of interest for the field of human nutrition/medicine if oleanolic acid is used as a nutraceutical, as the pig is a very good model for human nutritional physiology [23].

## 2. Materials and Methods

All research methods and procedures involving animals were conducted in accordance with the Spanish Policy on Protection of Animals used for Scientific Purposes (RD53/2013; Spain) and with European Union guidelines on protection of animals used for scientific research (Directive 2010/63/EU). The experimental protocol was reviewed by the Bioethical Committee of the Spanish Council for Scientific Research (CSIC, Spain) and the competent governmental authority (Junta de Andalucía, Spain, project reference 755/2018).

For HPLC-MS analysis, all chemicals were of analytical reagent grade or higher, whereas mobile phases were prepared with LC–MS grade solvents. Methanol, formic acid, acetic acid and ethanol were purchased from Fisher (Thermo Fisher Scientific, Leicestershire, UK). Purified water was obtained by a Milli-Q system from Millipore (Bedford, MA, USA). The standard for the oleanolic acid (98% purity) quantification was purchased from Sigma Aldrich (Steinheim, Germany).

### 2.1. Animals, Treatments and Diets

Twenty-four Landrace × Large White barrows (23.7 ± 1.0 kg body weight (BW)) obtained from a local supplier (Granja La Isla, Dúrcal, Granada, Spain) were randomly allocated in individual 2 m^2^ pens in an environmentally controlled room (21 ± 1.5 °C).

Three dietary treatments were used (8 growing pigs per treatment): Control (commercial diet without added oleanolic acid), OLA-1 (commercial diet supplemented with 260 mg/kg free oleanolic acid) and OLA-2 (commercial diet supplemented with 260 mg/kg cyclodextrin–oleanolic acid complex). Two microencapsulated oleanolic acid preparations obtained from olive leaves were provided by Innovaoleo S.L. (Córdoba, Spain): free oleanolic acid (not bound; 97.0%, *w*/*w*, purity) and cyclodextrin–oleanolic acid complex (21.8%, *w*/*w*, purity). These were used to prepare the OLA-1 and OLA-2 diets, respectively.

The control treatment was a standard diet (Table 1) for growing pigs (Cereales MACOB S.L. Villanueva Mesía, Granada, Spain), covering all nutrient requirements ([24]). The principal ingredients were corn, soybean meal, barley, wheat, lard, calcium carbonate, sodium chloride, calcium phosphate, l-lysine and mineral–vitamin premix (provided per kg of complete diet: 8000 IU vitamin A as retinyl acetate, 1600 IU vitamin D3 as cholecalciferol, 24 IU vitamin E as DL-*α*-tocopheryl acetate, 24 mg manganese as MnO, 57.6 mg iron as FeCO_3_, 80 mg zinc as ZnO, 440 μg I as KI, 80 mg of copper as CuSO_4_.5H_2_O and 400 μg cobalt as CoSO_4_.7H_2_O). Diets were supplemented with 0.3% Cr_2_O_3_ as an indigestible marker to determine digestibility.

Animals were randomly assigned to treatments and restrictively fed (85% ad libitum, on average) from the beginning. Water was freely available. Dietary intake was measured daily, taking spills into account. After one week of assay, pigs were fitted with chronic indwelling catheters in the jugular vein, as previously described [25], to carry out serial blood samplings. After recovery from the minor surgery (7 days), blood samples were collected into heparinized tubes (Monovette VetMed; Sarstedt, Nümbrecht, Germany) at 30, 60, 90, 120, 180 and 240 min after feeding of 500 g of the respective diets. Plasma samples were obtained (1820× *g*, 4 °C, 30 min; Eppendorf 5810 R, Hamburg, Germany) and frozen (−80 °C) pending subsequent oleanolic acid analysis.

On day 22 of the assay, the pigs were moved to metabolic crates and feces were collected for three days after two days of adaptation. Feces were pooled and frozen at −80 °C until analysis (dry matter, nitrogen, energy and oleanolic acid concentration).

Following an overnight fast, on day 31 of the assay, the pigs were weighed and then slaughtered (39.9 ± 2.43 kg BW) by electronarcosis and exsanguination. Blood was collected and plasma aliquots were frozen for biochemical analysis. Immediately after slaughter, ileal content was collected for oleanolic acid analysis and the empty gut and stomach, liver without gall bladder, heart, lungs, kidney, trachea, spleen, and visceral fat were extracted and weighed. Body weight gain, feed intake, and gain/feed ratio were used to determine performance.

### 2.2. Chemical Analysis

All analyses were performed in duplicate. Chemical analysis of the diets (Table 1) was performed using the standard procedures [26] for dry matter (No. 934.01) and ashes (No. 942.05). Nitrogen content was determined according to the Dumas procedure, using a LECO Truspec CN determinator (LECO Corporation, St Joseph, MI, USA), and crude protein was calculated as total nitrogen × 6.25. The gross energy content was determined using a PARR 1356 isoperibolic bomb calorimeter (Moline, IL, USA).

The apparent total tract digestibility of dry matter, organic matter, nitrogen and energy was obtained using the equation ATTD = 100 × [1 − (S*f*/Cr_2_O_3_*f*)/(S*d*/Cr_2_O_3_*d*)], where *f* and *d* subindices indicate concentrations in feces (or ileal content for apparent ileal digestibility) and diet, respectively. Chromium oxide in the diets, feces and ileal content was determined by a micromethod involving dry ashing and an alkaline fusion mixture [27].

Plasma metabolites and enzymes activities (creatinine, urea, total proteins, albumin, globulins, alkaline phosphatase, alanine transaminase, γ-glutamyl transferase, calcium and glucose) were determined by colorimetric assays using an automated Cobas Integra 400^®^ analyzer (Roche Diagnostics GmbH, Mannheim, Germany).

#### 2.2.1. Sample Preparation for Oleanolic Acid Analysis by HPLC-MS

Diets, freeze-dried ileal content and freeze dried feces were subjected to an optimized protocol of solid–liquid extraction. Ground sample (0.5 g) was mixed with aqueous methanol (5 mL; 20:80, *v*/*v*), vortexed for 10 s and sonicated for a period of 15 min in a refrigerated ultrasound bath. The supernatants were separated, and the solid residues were extracted again with the same procedure. The liquid fractions were combined together and evaporated in a Savant vacuum concentrator (Thermo Scientific, Waltham, MA, USA) at 35 °C. The dry residue was reconstituted with aqueous methanol, performing a centrifugation step (19,591× *g*, 10 min, 4 °C) to avoid solid particles in suspension, at a concentration adequate for quantification. The extraction was carried out in duplicate to assure the reproducibility of the process.

Plasma samples were deproteinized and concentrated prior to analysis by HPLC-MS. In brief, an aliquot of 100 μL of plasma was vortex-mixed with 150 μL of EtOH-MeOH (50:50, *v*/*v*) and maintained at −20 °C for 20 min. The mixture was centrifuged (19,591× *g*, 10 min, 4 °C) and the supernatant was evaporated in a vacuum concentrator for 3 h at ambient temperature. The samples were reconstituted in 50 μL of 95:5 (*v*/*v*) EtOH-water + 0.1% formic acid and centrifuged to avoid solid particles (19,591× *g*, 10 min, 4 °C). The supernatant was then transferred to HPLC vials.

#### 2.2.2. HPLC-MS Analysis of Oleanolic Acid

Samples prepared as described above were analyzed with a high-performance liquid chromatography instrument, Agilent 1260, coupled to an Agilent 6540 Ultra High Definition Accurate Mass Q-TOF analyzer by an ESI Jet Stream dual interface. The instrument was equipped with a micro vacuum degasser, a binary pump, an autosampler, a thermostat column and sample compartments, and a diode array detector. The selected stationary phase was a Zorbax Eclipse Plus C18 (150 × 4.6 mm, 1.8 μm particle size), whereas the mobile phases were water (mobile phase A) and methanol (mobile phase B), both acidified with 0.1% of formic acid. The mobile phase was pumped at 0.8 mL/min using the following gradient: 0 min, 5% B; 10 min, 75% B; 15 min, 100% B; 20 min, 100% B; 25 min, 5% B, and, finally, a conditioning cycle of 5 min at initial condition before the next injection. While the samples were maintained in a refrigerated state during the sequence run at 4 °C, a 5 μL aliquot was injected into the system and the separation was carried out at 30 °C. The optimized Q-TOF/MS parameters for the ionization source with a negative ionization mode were the following: 20 psig for nebulizing gas pressure, 10 L/min as dry gas flow, 12 L/min as sheath gas flow, dry and sheath gas temperature of 325 and 400 °C, respectively, and voltages of 4 and 0.5 kV for capillary and nozzle. The optimum ion transfer was achieved with voltages of 130, 45 and 750 V for fragmentor, skimmer and octopole 1 Rf Vpp. The acquisition was performed at a mass range of 100–1700 *m*/*z*, with acquisition rates of 3 spectra/second and 333.3 millisecond/spectrum. The calibration of the mass spectra was carried out with the continuous ionization of two reference ions: trifluoroacetic acid ammonium salt (*m*/*z* 112.9856) and hexakis (1H, 1H, 3H–tetrafluoropropoxy) phosphazine (*m*/*z* 1033.9881). Data acquisition in the centroid mode was performed by MassHunter Workstation software (Version B.06.01, Agilent Technologies, Palo Alto, CA, USA), whereas data analysis was carried out on MassHunter Qualitative Analysis B.06.00 (Agilent Technologies, Palo Alto, CA, USA).

For quantitative purposes, a stock solution of a commercial oleanolic acid standard was prepared at a concentration of 1000 mg/L in methanol. A six-level calibration curve (1–25 mg/L) was obtained by injecting in triplicate the oleanolic acid standard solution to obtain the equation y = 23,041x + 94,339, where y is the signal and x is the concentration.

The coefficient of determination R2 was 0.986. The limit of detection was estimated as the concentration of oleanolic acid in samples that generated a peak area at least 3 times higher than the baseline noise (0.016 ± 0.006 mg/L) and the limit of quantification was estimated as the concentration of oleanolic acid in samples that generated a peak area at least 10 times higher than the baseline noise (0.05 ± 0.02 mg/L).

The concentration of oleanolic acid in the samples was determined by interpolating the peak area measured in the chromatograms in the corresponding calibration curve of the commercial standard and expressed as μg/g for solid samples or μg/L for plasma.

### 2.3. Statistical Analysis

The number of animals (8/treatment, *n* = 24) was calculated using the G*Power software (Version 3.1, Heinrich-Heine-Universität Düsseldorf [28]), accepting an alpha risk of 0.05 and a beta risk of 0.2 in a two-sided test. For oleanolic acid concentration in plasma, repeated measures analyses were performed using the mixed procedure of SAS (PROC MIXED, SAS Institute Inc., Cary, NC, USA). The main effects in the model were the diet, the sampling time, and their interaction. As the effect of sampling time was not significant, it was withdrawn from the model. Initial BW was used as covariate for analysis of growth parameters and weight of organs. The experimental unit was the pig. Results were considered significant when *p* < 0.05 and as showing a tendency of significance with *p*-values between 0.05 and 0.10. Data were reported as least-squares means. For the rest of the parameters, the treatment effect was evaluated using the GLM procedure of SAS, which included the fixed effect of treatment (Control, OLA-1, OLA-2). Following analysis of variance, preplanned contrasts were generated using the contrast statement procedure of SAS (version 9.2; SAS Inst. Inc., Cary, NC, USA) to evaluate dietary treatment effect (Control vs. OLA-1, Control vs. OLA-2, and OLA-1 vs. OLA-2). In all of the cases, assumptions included a normal distribution of the data, equal variances, and randomization of the independent sample groups. In addition, normality and homogeneity of variances were checked with the Shapiro–Wilk test and Levene’s test, respectively.

## 3. Results

Growth parameters and viscera weights of pigs are shown in Table 2. Overall, growth and viscera weights were not affected (*p* > 0.05), except for decreased liver weight in pigs fed free oleanolic acid-supplemented diets, compared with the other diets (*p* < 0.05).

Apparent total tract digestibility (ATTD) of nutrients and oleanolic acid and apparent ileal digestibility (AID) of oleanolic acid in growing pigs fed the control diet and diets supplemented with unbound oleanolic acid and cyclodextrin-bound oleanolic acid are shown in Table 3. No differences in the digestibility of oleanolic acid were found at the fecal or ileal level, except for an increase in ATTD of oleanolic acid in OLA-1 compared to Control treatment (8.7%; *p* < 0.05). Pigs fed the OLA-1 diet had decreased ATTD of energy (2%; *p* < 0.05) and showed a trend for decreased ATTD of dry matter (0.05 < *p* < 0.10), compared with control pigs. Pigs fed OLA-2 diet had increased ATTD of organic matter compared with control pigs (2.3%; *p* < 0.05). Furthermore, when OLA-1 and OLA-2 treatments were compared, increased ATTD of dry matter, organic matter and energy was found in pigs supplemented with cyclodextrin-bound oleanolic acid (2.6–3.0%; 0.01 < *p* < 0.05).

The plasma biochemical parameters of pigs are shown in Table 4. Overall, no differences were found among treatments (*p* > 0.05), except for increased plasma calcium and alkaline phosphatase in pigs fed Free-OLA diets (*p* < 0.05) and a trend to increased alkaline phosphatase in OLA-1 compared to OLA-2 pigs. Increased plasma oleanolic acid was found in both pigs fed OLA-1 (*p* < 0.05) and pigs fed OLA-2 (0.05 < *p* < 0.10), compared to control pigs. The original data can be found in Table S1.

## 4. Discussion

According to the available literature, a variety of strategies have been used to improve the solubility of oleanolic acid and hence its oral bioavailability [13,19,20,21,22]. In the present study, microencapsulated oleanolic acid, either in its free form or complexed to cyclodextrin, was included in the compound matrix of a pig diet in an attempt to improve the solubility of the oleanolic acid. In this sense, cyclodextrins have previously been used to promote water solubility of triterpenes [13]. Although a study on the digestibility of oleanolic acid has been carried out in vitro [29] using the INFOGEST protocol, to the best of our knowledge, there is no information in the literature about in vivo digestibility of oleanolic acid in humans or animals. The elevated digestibility of both oleanolic acid preparations in the present experiment contrasts with the corresponding plasma levels and the reported low oral bioavailability in other animals, as evaluated by the appearance in systemic circulation [15]. The digestibility of oleanolic acid is certainly an initial estimation of oral bioavailability, as foods must undergo digestion before reaching the small intestine for absorption. Likewise, the rest of components of the diet (protein, carbohydrates, fiber and fat) may interact with oleanolic acid, thereby altering its absorption; digestibility depends on the composition of the diet. In the present experiment, oleanolic acid digestibility was evaluated both at the ileum level (apparent ileal digestibility, AID), to avoid, as much as possible, the influence of microbiota, and at the fecal level (apparent total tract digestibility, ATTD) using an inert indigestible marker. It was assumed that no endogenous secretion of oleanolic acid to the gut occurred. Ileal digestibility of oleanolic acid was higher compared to total tract digestibility in the present experiment, probably indicating that microbiota was metabolizing oleanolic acid reaching the hindgut. Passive diffusion seems to be the main mechanism of oleanolic acid transport through the gut barrier, with the apparent permeability coefficient indicating moderate absorption across intestinal cells, according to model studies on Caco-2 cells [15]. On the other hand, the appearance in systemic blood of oleanolic acid after dietary intake proved that enterocytes absorbed the molecule, although no major differences between the two oleanolic acid preparations were noticed. It must be pointed out that the pigs were given to consume a lesser amount of oleanolic acid (8.8 mg oleanolic acid/kg BW) than used in rodent studies (10–300 mg oleanolic acid/kg BW [16,20,21]. Thus, the concentration levels for some of the plasma samples were below the limit of quantitation in spite of the high sensitivity of the analytical method used for the analysis. The greatest peak of oleanolic acid in plasma occurred between 3 and 4 h postprandial, compared with the 0.25–2.75 h reported in rats [16,20,21] or the 1.5 h in beagle dogs [30]. Maximum oleanolic acid plasma concentration (77 µg/L) was within the lower range of values reported for rats orally fed oleanolic acid preparations (60–810 µg/L [31]. Although the digestibility of oleanolic acid was elevated, the low level of its appearance in systemic blood could be explained by the restricted amount of oleanolic acid in the diets and by the extensive metabolism by cytochrome P450 at the intestine and liver level [17,32].

The large variation of plasma oleanolic acid concentration in studies with rats, dogs and humans, as well as in the present experiment, may be due to the low oral absorption, individual variations [20], differences between species, or the oleanolic acid being administered as a pure compound or in a complex matrix such as a diet. As expected, the values obtained were lower than those reported in rats and humans administered different forms of oleanolic acid at greater concentrations than in the present experiment [33,34], in which no differences could be found between free and cyclodextrin-bound oleanolic acid.

To the best of our knowledge, there is no information regarding the effects of oleanolic acid intake on growth, nutrient digestibility and viscera weight in pigs. In the present study, oleanolic acid did not significantly alter the body weight of growing pigs after a 4-week growth trial. Similar results were found after short-term treatment (10 days) with oleanolic acid in mice [5] and rats [35]. On the contrary, decreased body weight was reported after oleanolic acid treatment (intraperitoneal or oral) in several different models of overweight rodents: obese diabetic mice [2,7], rats with metabolic dysfunctions induced by a high-fat—high-fructose diet [36], rats fed high-fat diets [37] and prediabetic rats [38].

Regarding organ weights, liver weight of pigs fed OLA-1 decreased compared to Control and OLA-2 pigs. Decreased liver weight has been reported as well in rats fed high-fat diets supplemented with oleanolic acid for four weeks [37], prediabetic rats fed oleanolic acid for twelve weeks [38], diabetic mice treated intraperitoneally with oleanolic acid for two weeks [7] and obese mice fed oleanolic acid for sixteen weeks [2]; this effect has been explained by the reduced accumulation of lipids in the hepatocytes induced by supplementation. Visceral fat and the rest of the organs were not affected in pigs. On the contrary, administration of this compound decreased visceral fat in rats fed high-fat diets for four weeks [37], prediabetic rats fed oleanolic acid for twelve weeks [38], mice fed a high-fat diet [39], obese mice fed oleanolic acid for sixteen weeks [2] and diabetic mice treated intraperitoneally with oleanolic acid for two weeks [7].

As far as we know, there is no information regarding the effect of oleanolic acid on nutrient digestibility in vitro or in vivo. In growing pigs, the effect of oleanolic acid on nutrient digestibility was small in magnitude, with increases in dry matter and organic matter ATTD when oleanolic acid was administered bound to cyclodextrin, while decreases were seen for energy and dry matter ATTD when it was provided in its free form. Interestingly, cyclodextrins have been reported to decrease nutrient digestibility in dogs [40] and humans [41]. According to our results, ATTD was increased, relative to free oleanolic acid, when oleanolic acid was administered bound to cyclodextrin, which in principle should increase the bioavailability of oleanolic acid. Nevertheless, this increased digestibility of cyclodextrin-bound oleanolic acid did not translate into greater plasma concentration.

Although it has been reported that oleanolic acid has antidiabetic properties in mice [2,7,11], no changes in glucose concentration were detected in the present study, which used non-diabetic pigs. In addition, it has been demonstrated that oleanolic acid is highly bound to albumin in human [42] and rat [16] sera, facilitating its distribution to the different tissues and organs. However, no differences in plasma albumin concentration were found in the present research which indicated a similar distribution of oleanolic acid in OLA-1 and OLA-2 pigs.

Interestingly, calcium concentration and alkaline phosphatase activity were greater in OLA-1, compared to Control pigs, which may indicate an effect on bone mineralization [43]. In line with this, the roles of oleanolic acid in protecting against bone loss, increasing calcium balance and modulating vitamin D metabolism in aged rats fed a mixture of oleanolic and ursolic acid have been previously reported [22]. Nevertheless, further studies need to be carried out to confirm the effect of oleanolic acid on bone metabolism.

One limitation of our study is the relatively reduced number of animals used, particularly for the growth parameters. Additionally, it would be interesting to extend our study to other phases of growth such as piglets and finishing pigs, as well as gilts and sows.

On the other hand, the results can be of interest in the field of human nutrition, as the pig is an excellent model for translational research.

## 5. Conclusions

The present results indicated that dietary oleanolic acid was well digested in growing pigs, while the bioavailability of the studied formulations seem to be low, with relatively small amounts of oleanolic acid in systemic blood. Although consumption of oleanolic acid slightly influenced digestibility of energy and organic matter, growth parameters were not affected. Moreover, alterations in biochemical parameters suggest an effect of oleanolic acid on bone metabolism.

## Figures and Tables

**Table 1:** Analyzed chemical composition of the diets, g/kg, on an as-is basis.

	Control	OLA-1	OLA-2
Dry matter	896.3	895.5	894.7
Ashes	46.7	47.5	47.7
Crude protein	161.4	164.3	163.4
Ether extract	41.8	38.2	39.2
Gross energy (MJ/kg)	16.6	16.7	16.7
Oleanolic acid	32	260	260

**Table 2:** Growth parameters and viscera weight in growing pigs (*n* = 8) fed unsupplemented diets (Control), diets supplemented with free oleanolic acid (OLA-1) and diets supplemented with cyclodextrin–oleanolic acid complex (OLA-2).

	Control	OLA-1	OLA-2	SEM	Contrasts		
					Control vs. OLA-1	Control vs. OLA-2	OLA-1 vs. OLA-2
Initial body weight, kg	23.7	23.7	23.7	1.01	0.999	0.962	0.968
Average daily gain, kg/d	0.418	0.466	0.462	0.040	0.940	0.832	0.940
Intake, g/d	1042	1064	1071	44.7	0.708	0.594	0.758
Gain/feed	0.393	0.433	0.429	0.024	0.940	0.497	0.883
Final body weight, kg	38.8	40.5	40.3	2.43	0.634	0.634	0.945
Carcass, kg	29.6	29.4	29.9	1.53	0.844	0.890	0.735
Liver, g	1029	832	1049	43.2	0.008	0.762	0.022
Kidneys, g	217	189	213	11.1	0.191	0.840	0.088
Stomach, g	345	326	349	18.5	0.526	0.887	0.433
Blood, g	1745	1682	1674	96	0.621	0.624	0.963
Heart, g	221	192	220	9.0	0.505	0.942	0.470
Spleen, g	109	95	94	9.1	0.340	0.310	0.898
Lungs and trachea, g	933	1021	1001	75	0.607	0.832	0.459
Visceral fat, g	369	373	442	90	0.970	0.570	0.540

**Table 3:** Apparent total tract digestibility of oleanolic acid and nutrients and apparent ileal digestibility (AID) of oleanolic acid in growing pigs fed unsupplemented diets (Control), diets supplemented with free oleanolic acid (OLA-1), or diets supplemented with cyclodextrin–oleanolic acid complex (OLA-2) (*n* = 8).

	Control	OLA-1	OLA-2	SEM	Contrasts		
					Control vs. OLA-1	Control vs. OLA-2	OLA-1 vs. OLA2
Dry matter	0.801	0.789	0.811	0.004	0.069	0.110	0.008
Organic matter	0.817	0.811	0.836	0.004	0.283	0.039	0.014
Nitrogen	0.831	0.817	0.836	0.005	0.141	0.428	0.243
Energy	0.829	0.812	0.833	0.004	0.008	0.587	0.009
OLA ^1^	0.748	0.819	0.756	0.030	0.014	0.875	0.197
OLA AID ^2^	0.856	0.899	0.866	0.034	0.435	0.862	0.445

^1^ Apparent total tract digestibility of oleanolic acid. ^2^ Apparent ileal digestibility of oleanolic acid.

**Table 4:** Biochemical parameters of plasma in growing pigs fed a control diet, diets supplemented with unbound oleanolic acid, or diets supplemented with cyclodextrin-bound oleanolic acid (*n* = 8).

	Control	OLA-1	OLA-2	SEM	Contrasts		
					Control vs. OLA-1	Control vs. OLA-2	OLA-1 vs. OLA-2
Albumin, g/L	30.7	31.0	32.3	2.18	0.917	0.611	0.661
Alkaline Phosphatase, U/L	40	75	40	11.6	0.052	0.970	0.060
Alanine transaminase, U/L	27.5	33.3	32.4	3.53	0.302	0.342	0.852
Calcium, mg/dL	0.539	0.805	0.638	0.06	0.016	0.140	0.146
Creatinine, mg/dL	1.29	1.29	1.30	0.070	0.949	0.916	0.960
γ-glutamyltransferase, U/L	57.4	60.7	71.0	7.84	0.776	0.288	0.331
Globulins, g/L	36.5	37.8	37.8	2.45	0.503	0.482	0.993
Glucose, mg/dL	198	193	195	21.3	0.848	0.929	0.931
Total proteins, g/L	67.1	65.3	66.5	2.39	0.623	0.853	0.654
Urea N, mg/dL	21.4	22.3	25.8	2.01	0.776	0.111	0.253
Oleanolic acid, µg/L	0.036	5.43	3.15	1.570	0.023	0.083	0.389

## Data Availability

The data presented in this study are available on request from the corresponding author.

## Supplementary Materials

The following are available online at https://www.mdpi.com/article/10.3390/ani14192826/s1, Table S1: original data.
